# Small-Size Graphene-Enabled Corrosion-Resistant Ultra-Thin Copper Foils Fabricated by Direct-Current Electrodeposition

**DOI:** 10.3390/molecules31183341

**Published:** 2026-09-20

**Authors:** Zheng Yu, Hanyang Zhao, Junpeng Li, Jinzhong Li, Lixu Zhu, Xiaowen Zhang, Wenjuan Niu, Feng Wang, Kewang Zheng, Fei Zhong

**Affiliations:** 1 School of Chemistry and Materials Science, Hubei Engineering University, Xiaogan 432000, China; 2 Key Laboratory of Agricultural Equipment in Mid-Lower Yangtze River, Ministry of Agriculture, College of Engineering, Huazhong Agricultural University, Wuhan 430070, China; 3 Dongguan Yichuang Surface Treatment Technology Co., Ltd., Dongguan 523000, China

**Keywords:** copper foil, graphene, electrolytic deposition, corrosion resistance, electrochemical behavior

## Abstract

The corrosion degradation of ultra-thin copper foils remains a critical challenge for maintaining their long-term structural stability under aggressive electrochemical environments. In this work, small-size graphene (sGr) was incorporated into ultra-thin copper foils through a direct-current electrodeposition strategy to improve corrosion resistance. Compared with conventional graphene, the reduced graphene size facilitated a more uniform dispersion within the copper matrix and effectively regulated the surface morphology and crystallographic texture of the deposited copper foils. The resulting sGr/Cu composite foil exhibited reduced surface defects, enhanced (220) preferred orientation with a texture coefficient of 83.7%, and improved electrochemical corrosion resistance in 3.5 wt.% NaCl solution. Specifically, the sGr/Cu foil showed a more positive corrosion potential (−0.099 V), a lower corrosion current density (3.781 × 10^−5^ A·cm^−2^), and a higher charge transfer resistance (3789 Ω·cm^2^) compared with pure copper foil. The enhanced corrosion resistance was attributed to the synergistic effects of improved graphene dispersion, graphene-induced barrier effects, surface morphology optimization, and strengthened graphene/copper interfacial interactions. X-ray photoelectron spectroscopy analysis suggested the presence of Cu–O–C-related interfacial interactions, which may contribute to improved interfacial stability and corrosion protection. This work provides an effective strategy for designing corrosion-resistant ultra-thin copper foils through graphene size regulation and interface engineering.

## 1. Introduction

Copper foil plays a critical role in lithium-ion batteries (LIBs). Serving as the current collector for the negative electrode, copper foil directly influences battery efficiency, cycle life, and safety through its surface state and interfacial stability [1,2,3]. However, during prolonged charge–discharge cycling, decomposition products of LiPF_6_ in the electrolyte, such as HF, together with trace moisture, can chemically attack ultra-thin copper foils (6–12 μm). Such chemical attacks induce electrochemical corrosion and oxidation of copper foils. Corrosion not only generates a high-impedance corrosion product layer on the copper foil surface, thereby increasing interfacial resistance, but also weakens the adhesion between the copper foil and active materials. This weakened interface can cause active material detachment, thereby accelerating capacity degradation and compromising battery safety [4,5,6]. Consequently, enhancing the corrosion resistance of ultra-thin copper foils under electrolyte environments has become essential for improving their long-term stability and reliability.

Introducing reinforcement phases is an effective strategy for improving the corrosion resistance of copper foils. The commonly used reinforcement phases for copper foils can be mainly classified into two categories: cermets and carbon nanomaterials, such as carbon nanotubes (CNTs) and graphene (Gr) [7,8]. Although cermet reinforcements can improve certain properties of copper foils, their incorporation may reduce electrical conductivity, which is a critical requirement for copper current collectors [9]. In contrast, carbon nanomaterials exhibit excellent electrical conductivity, chemical stability, and corrosion resistance. Among these carbon nanomaterials, graphene nanoplatelets can form physical barriers on copper foil surfaces or along grain boundaries, effectively hindering the diffusion of corrosive species, such as Cl^−^ and HF, and thereby suppressing corrosion processes [10,11]. Therefore, incorporating carbon nanomaterials into copper foils represents an effective approach for enhancing corrosion resistance.

Considerable progress has been achieved in the development of graphene-reinforced copper matrix composites. However, challenges remain in fabricating graphene/copper composite foils with uniform graphene distribution and enhanced corrosion resistance. First, existing fabrication methods for Cu/Gr composites still suffer from several limitations [12,13]. For example, powder metallurgy is generally unsuitable for the cost-effective fabrication of composite foils with thicknesses below 10 μm, while chemical vapor deposition (CVD) faces challenges in controlling the uniformity of three-dimensional graphene/copper structures, thereby limiting the barrier effect of graphene against corrosive species. Second, graphene tends to agglomerate due to strong van der Waals interactions between graphene layers and preferentially accumulates along grain boundaries within the copper matrix [14,15]. This aggregation not only reduces the effectiveness of graphene as a physical barrier but may also introduce interfacial micro-gaps or preferential corrosion pathways along grain boundaries, thereby accelerating localized corrosion.

To address these challenges, this study introduces size-reduced graphene into copper foils via a direct-current (DC) electrodeposition method. By optimizing the electrodeposition parameters and composite additives, the dispersion of graphene within the copper matrix was improved, enabling the fabrication of size-reduced graphene/copper composite foils (sGr/Cu). The microstructure and corrosion behavior of the sGr/Cu foils were systematically investigated in 3.5 wt.% NaCl solution. The corrosion protection mechanism was further elucidated based on multi-scale characterization. This study aims to develop corrosion-resistant graphene-reinforced copper foils and provide insights into the role of graphene size regulation and interfacial optimization in improving corrosion resistance.

## 2. Results

### 2.1. Microstructural Morphology and Analysis

**Figure 1:**
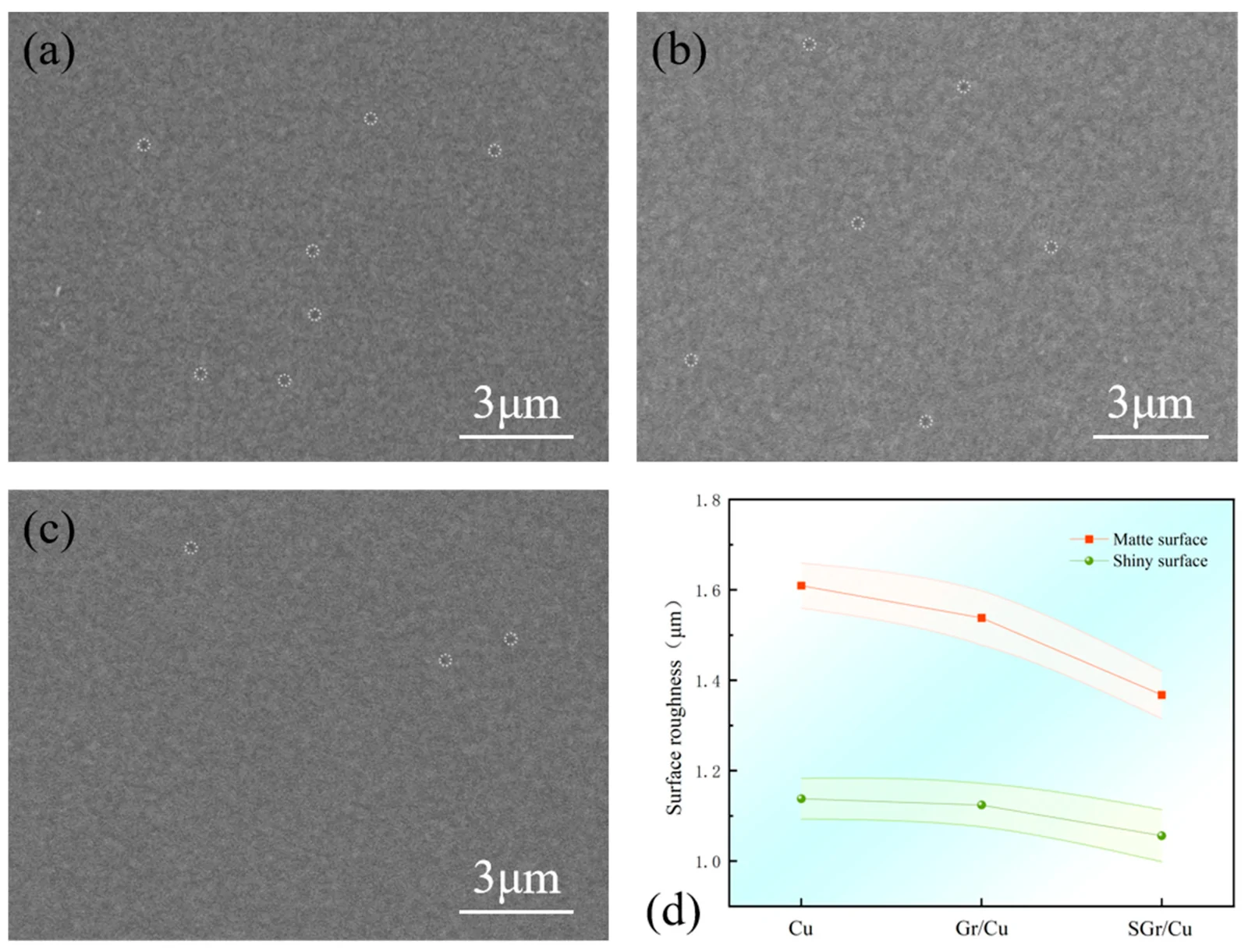
(**a**–**c**) SEM images of the matte surface of Cu, Gr/Cu, and sGr/Cu foils; (**d**) Surface roughness (Rz) values.

Figure 1a–c present the SEM images of the matte surfaces of the three copper foil samples. The overall macroscopic surface morphologies of the three samples exhibit only minor differences. The copper grains are densely packed and uniformly distributed, without obvious large-area pits or cracks. This relatively uniform surface morphology provides a favorable basis for the corrosion resistance of the copper foil. However, differences can be observed in the local surface features of the three samples. A small number of surface defects are observed on the matte surfaces of all three samples (marked by white dashed lines). These defects may serve as preferential sites for the adsorption and accumulation of corrosive species. Notably, fewer surface defects are observed after introducing size-reduced graphene, accompanied by a smoother and more compact surface morphology. Ball-milling reduces the lateral size of graphene sheets (Figure S1), weakens van der Waals interactions between graphene layers, and improves the dispersion stability of graphene in the plating electrolyte. Accordingly, small-size graphene sheets can be co-deposited more homogeneously on the cathode, modulate the nucleation sites of copper ions, suppress the abnormal growth of copper grains, and consequently realize more uniform co-deposition between graphene and the copper matrix.

The surface roughness results in Figure 1d further quantify these morphological differences. The Rz values of the matte surfaces for Cu, Gr/Cu, and sGr/Cu are 1.610 μm, 1.538 μm, and 1.368 μm, respectively. The corresponding shiny-side roughness values are 1.138 μm, 1.124 μm, and 1.056 μm, respectively. Among the three samples, sGr/Cu exhibits the lowest surface roughness, consistent with the SEM observations. A smoother surface can reduce the retention of corrosive electrolytes and localized surface attack, while also providing a more uniform substrate for subsequent surface processing.

### 2.2. X–Ray Diffraction Analysis

**Figure 2:**
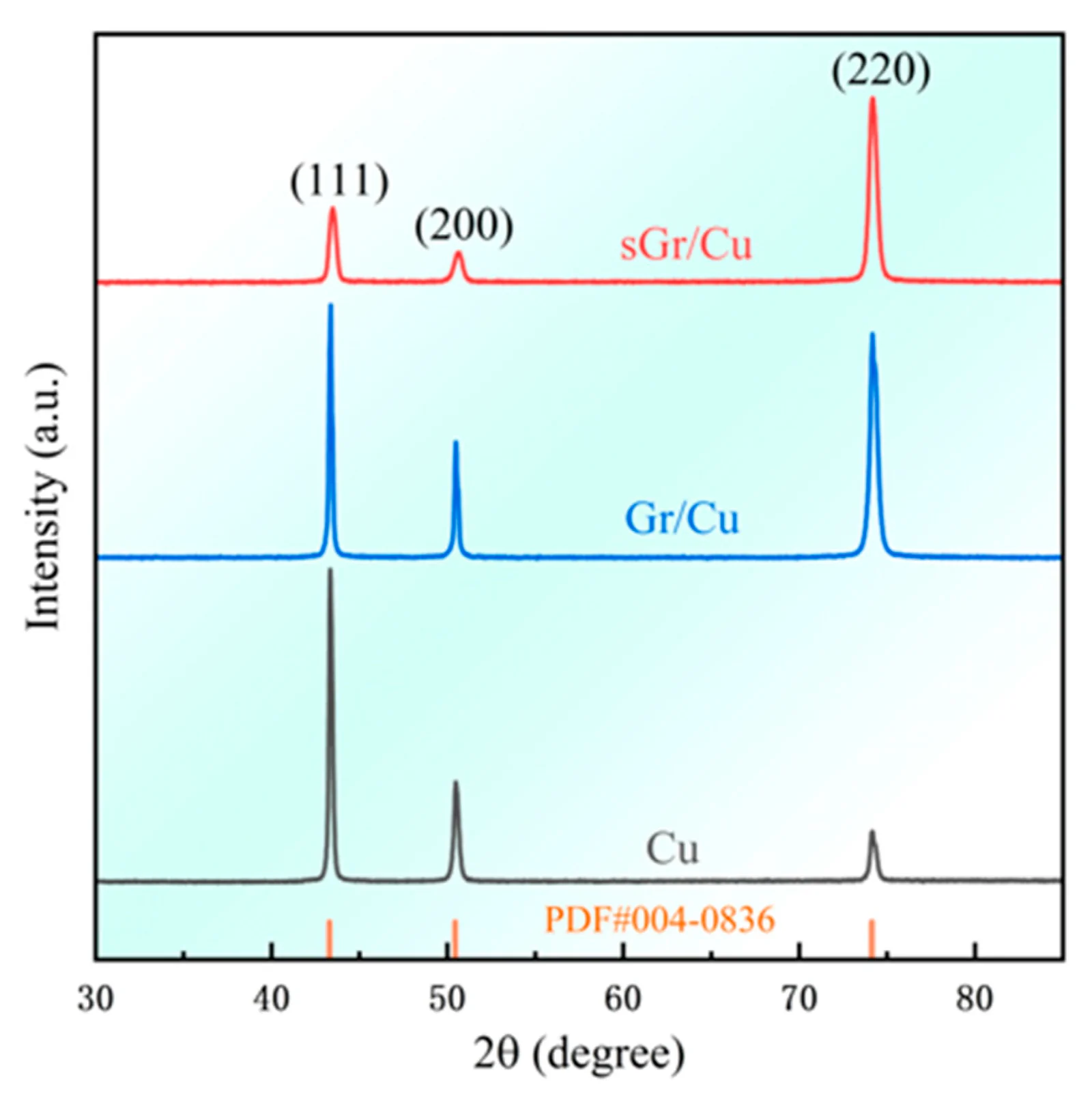
XRD patterns.

Figure 2 presents the XRD patterns of the three copper foil samples. The diffraction peaks of all samples correspond well with the face-centered cubic (FCC) copper phase (PDF#04–0836), indicating that the prepared copper foils retain the FCC crystal structure without detectable impurity phases [16].

To quantitatively evaluate the influence of graphene size on copper texture, texture coefficients (TCs) for different crystallographic planes were calculated according to Equation (1) [17], and the results are summarized in Table 1. The pure copper foil exhibits texture coefficients of 36.6%, 27.6%, and 35.8% for the (111), (200), and (220) planes, respectively. The relatively similar values indicate the absence of a pronounced preferred orientation, suggesting relatively random grain growth in the absence of graphene. After introducing pristine graphene, the (111) texture coefficient of the Gr/Cu foil decreases to 15.3%, whereas the (220) texture coefficient increases to 68.2%, indicating the development of a stronger (220) preferred orientation. This result is consistent with previous reports that graphene incorporation can modify the nucleation and growth kinetics of copper during electrodeposition. Specifically, graphene sheets adsorbed on the cathode surface may influence the deposition rate of copper ions on different crystal planes, thereby altering the competitive growth behavior among crystallographic orientations [18]. More importantly, with the introduction of size-reduced graphene, the (220) texture coefficient of the sGr/Cu foil further increases to 83.7%, while the (111) and (200) coefficients decrease to 7.6% and 8.7%, respectively. This suggests that ball-milled size-reduced graphene exhibits a stronger ability to regulate copper texture and promote preferential growth of the (220) plane. Previous studies have reported a correlation between the (220) preferred orientation of copper foils and their corrosion resistance. For example, Song et al. investigated the corrosion behavior of copper foils with different textures and found that (220)-oriented copper foils exhibited improved corrosion resistance in chloride-containing environments, which was attributed to their different surface energy characteristics and atomic arrangements [19]. Therefore, the enhanced (220) texture in the sGr/Cu foil may contribute to its improved corrosion resistance observed in subsequent electrochemical tests.

(1) $$T_{c(hkl)}=\frac{I_{\left(hkl\right)}/I_{0(hkl)}}{\sum I_{\left(hkl\right)}/I_{0(hkl)}}\times 100\%$$

where *I*_(*hkl*)_ represents the experimental intensity of the chosen (hkl) plane, and *I*_0(*hkl*)_ is the standard intensity of the same plane in the JCPDS cards.

### 2.3. Raman Analysis

**Figure 3:**
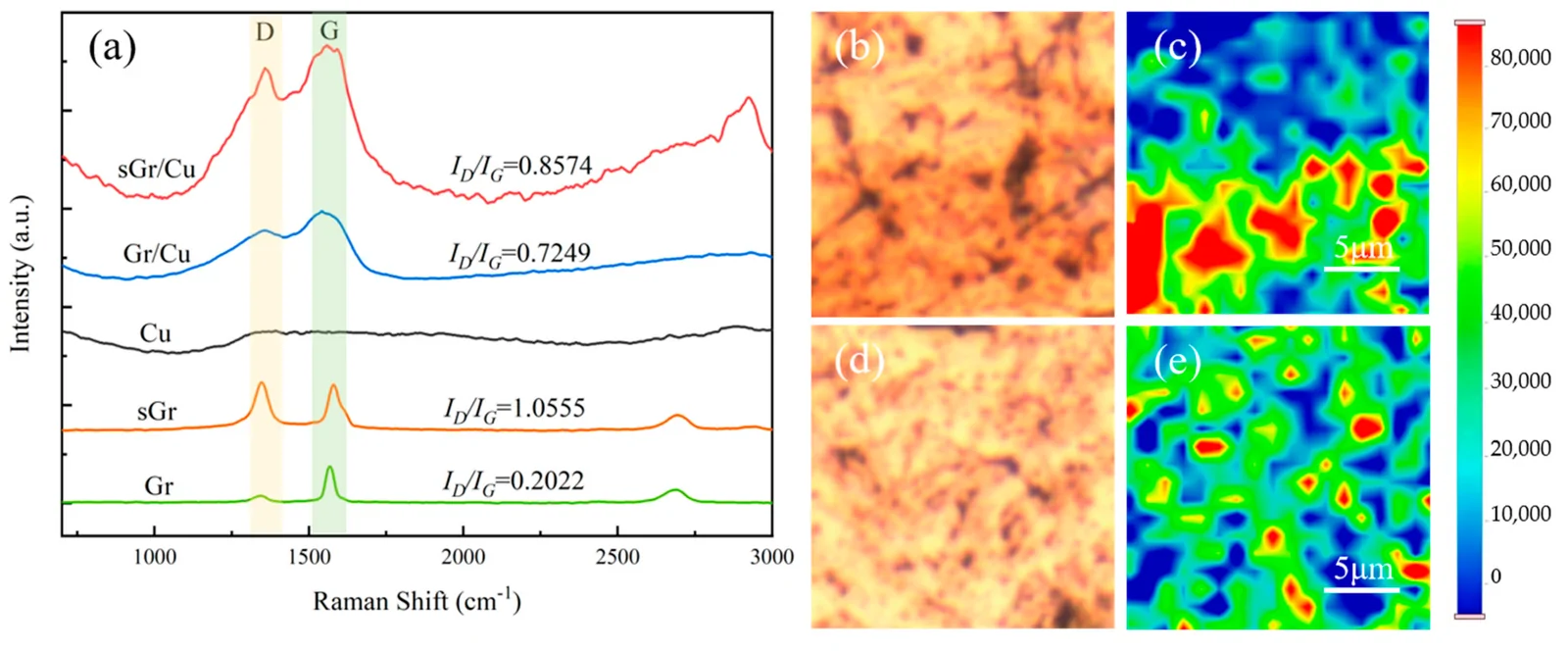
(**a**) Raman spectra; (**b**,**c**) Raman mapping images of Gr/Cu; (**d**,**e**) Raman mapping images of sGr/Cu.

To further confirm the presence, state, defect level, and spatial dispersion uniformity of graphene in the Cu matrix, Raman spectroscopy and Raman mapping analyses were performed on the samples, and the results are shown in Figure 3. As can be seen from Figure 3a, no obvious characteristic D and G bands of carbon materials are observed for the pure Cu sample; both pristine Gr and ball-milled sGr exhibit obvious D and G bands at approximately 1350 cm^−1^ and 1580 cm^−1^, respectively; after incorporation, Gr/Cu and sGr/Cu also retain the characteristic peaks of graphene, indicating that graphene has been successfully co-deposited into the Cu matrix.

Previous studies have demonstrated that both zero-dimensional point defects and one-dimensional line defects can activate the D-band signal in graphene-related materials [20,21,22]. The pristine Gr sample shows an *I_D_*/*I_G_* value of only 0.2022, indicative of a relatively intact sp^2^ lattice with low intrinsic disorder. After 50 h of planetary ball-milling, the *I_D_*/*I_G_* ratio of sGr increases to 1.0555. Although ball-milling introduces basal-plane point defects, the rise in *I_D_*/*I_G_* does not exclusively originate from in-plane point-like defects2. When the lateral domain size of graphene decreases, the increased edge density can also raise this ratio, which has been comprehensively discussed in well-established disorder models for graphitic carbons. Accordingly, the elevated *I_D_*/*I_G_* of ball-milled sGr stems from the combined effect of newly generated in-plane defects and abundant flake edges. TEM characterization (Figure S1) further confirms the reduced lateral dimension and abundant edge sites of ball-milled sGr. After co-electrodeposition with copper, the sGr/Cu composite delivers an *I_D_*/*I_G_* of 0.8574, still higher than the value of 0.7249 for Gr/Cu. This observation can be attributed to the smaller sheet size and richer edge sites of sGr, as well as the more sufficient interfacial contact between graphene sheets and the copper matrix.

Raman mapping further reveals the spatial distribution uniformity of graphene in the Cu matrix [23]. Figure 3b–e show the Raman signal intensity mappings of Gr/Cu and sGr/Cu. By comparison, the high-intensity regions in Gr/Cu exhibit a relatively large block-like distribution, with obvious local enrichment or aggregation; in contrast, the high-intensity regions in sGr/Cu are smaller in size and more uniformly distributed, without large-area continuous enrichment. This indicates that after ball milling reduces the graphene size, its dispersion stability in the electrolyte is improved, allowing more uniform co-deposition with the Cu matrix during electrodeposition. This result is consistent with the SEM observations that sGr/Cu exhibits a smoother surface, fewer surface defects, and lower surface roughness.

### 2.4. Corrosion Morphology and Corrosion Product Analysis

**Figure 4:**
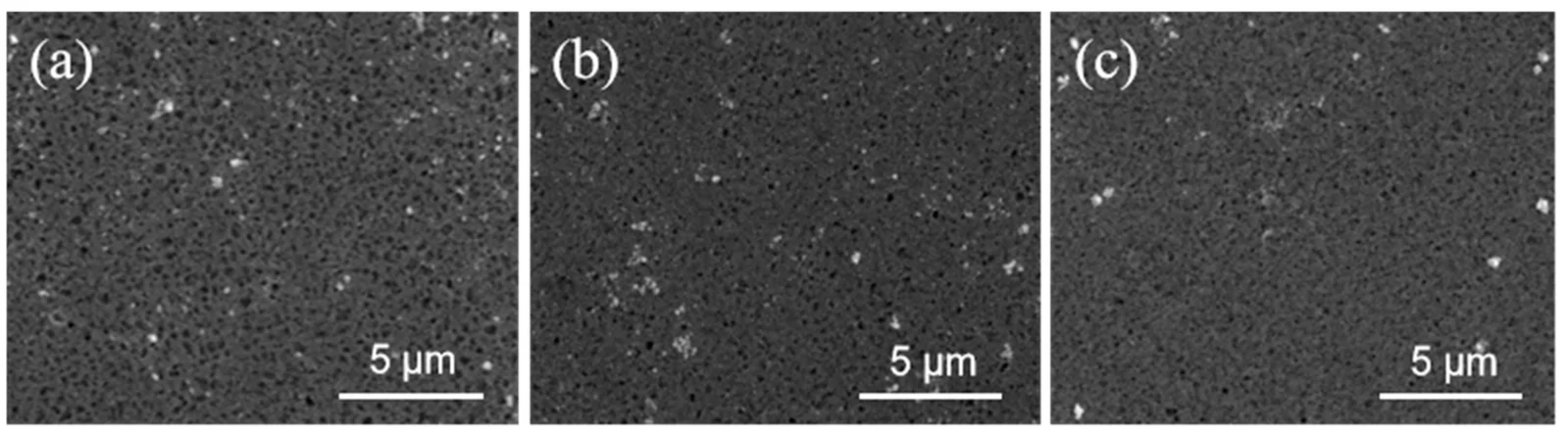
SEM images showing the surface corrosion morphology of (**a**) Cu, (**b**) Gr/Cu, and (**c**) sGr/Cu foils after immersion in 3.5 wt.% NaCl solution for 48 h.

Figure 4a–c present the surface corrosion morphologies of the three copper foils after immersion in 3.5 wt.% NaCl solution for 48 h. The surface of pure copper foil is covered with numerous large and deep circular pits, indicating severe localized pitting corrosion. The number of corrosion pits on the Gr/Cu foil decreases, although localized accumulation of corrosion products remains visible. In contrast, the sGr/Cu composite foil exhibits only a few small and shallow corrosion pits without interconnected corrosion regions. The corrosion products are relatively small and sparsely distributed, suggesting a lower degree of corrosion attack. Quantitative statistics of corrosion pits in Table S1 further confirm that the average pit diameter decreases sequentially for Cu, Gr/Cu and sGr/Cu samples, and the areal density of corrosion pits (area fraction) drops from 9.724% to 4.872% and 3.138%, respectively, demonstrating that the introduction of small-size graphene can effectively suppress the initiation and growth of pitting corrosion.

For the pristine graphene composite, weak graphene/copper interfacial bonding may provide preferential pathways for Cl^−^ ions penetration along interfacial gaps, generating localized corrosion environments that accelerate corrosion attack [24,25]. In the sGr/Cu composite, size-reduced graphene is more uniformly dispersed within the copper matrix, reducing the formation of continuous interfacial gaps. The sheet-like structure of graphene may establish a tortuous physical barrier within the copper matrix, increasing the diffusion resistance of Cl^−^ ions and water molecules and reducing the penetration of corrosive species. Furthermore, the reduced surface roughness of sGr/Cu may decrease the adsorption and retention of corrosive species on the surface. The synergistic effects of graphene dispersion and surface morphology optimization may suppress the initiation and propagation of pitting corrosion, supporting the effectiveness of the size-reduced graphene dispersion strategy for improving the corrosion resistance of ultra-thin copper foils.

### 2.5. Electrochemical Corrosion Behavior

**Figure 5:**
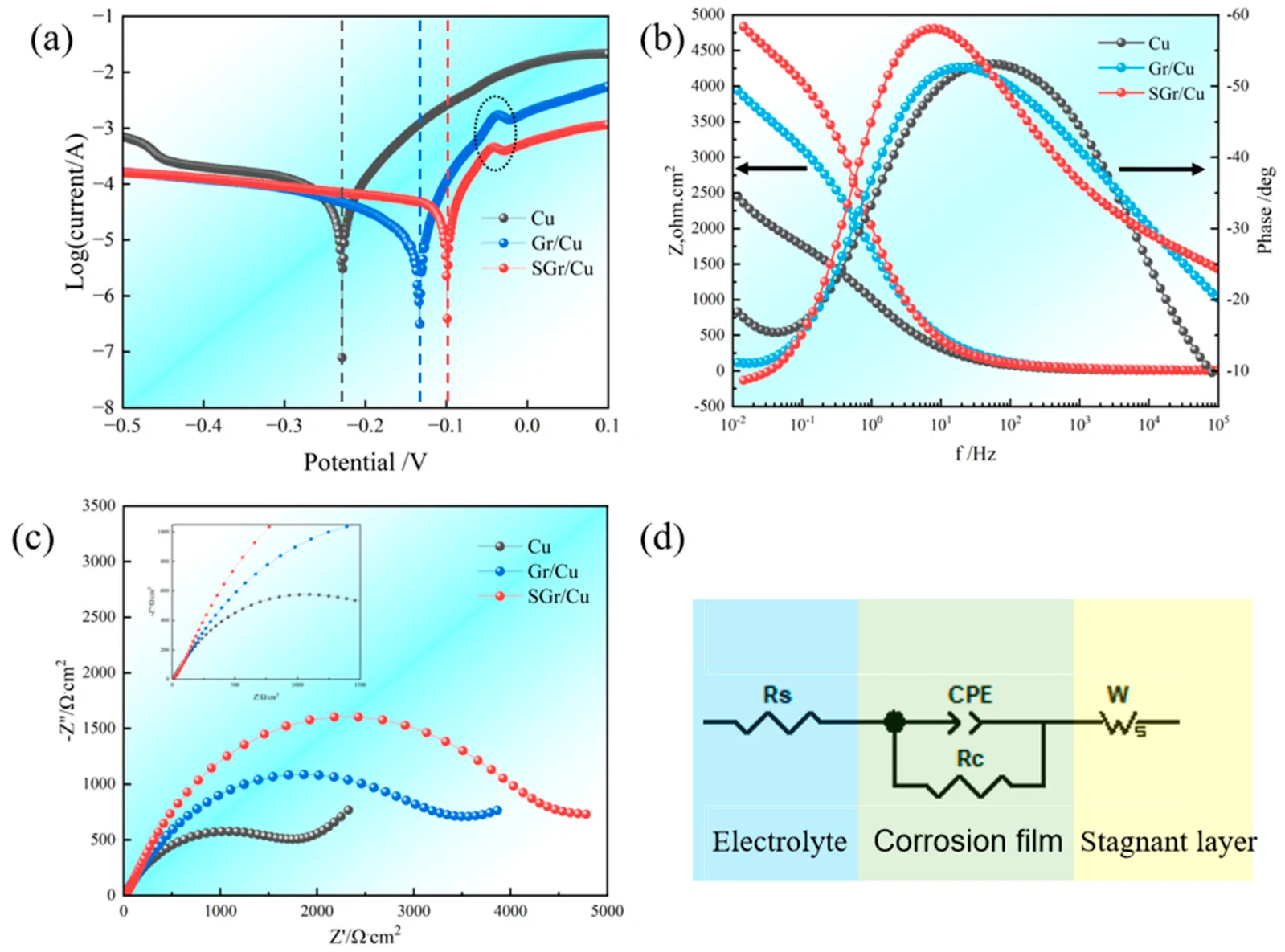
(**a**) Tafel polarization curves, (**b**) Bode plots, (**c**) Nyquist plots, (**d**) Equivalent electrical.

Figure 5a presents the Tafel polarization curves of the three samples, and the corresponding fitted corrosion parameters are summarized in Table 2. For the Cu sample, the corrosion potential (Ecorr) is −0.229 V, with a corrosion current density (icorr) of 9.594 × 10^−5^ A·cm^−2^. For Gr/Cu, Ecorr shifts positively to −0.133 V, while icorr decreases to 4.275 × 10^−5^ A·cm^−2^. The sGr/Cu composite foil exhibits a further positive shift in Ecorr to −0.099 V and the lowest icorr value of 3.781 × 10^−5^ A·cm^−2^. The positive shift in corrosion potential suggests a lower corrosion tendency, while the decreased corrosion current density indicates suppressed corrosion kinetics. Notably, in the anodic region, both Gr/Cu and sGr/Cu polarization curves exhibit a passivation region (indicated by the dashed box). The improved dispersion of graphene may establish a more effective physical barrier within the copper matrix, reducing direct contact between corrosive species and the copper matrix [26,27]. Although pristine graphene also provides a barrier effect, its stronger aggregation tendency may generate local corrosion pathways at graphene/copper interfaces. Therefore, the corrosion resistance improvement of Gr/Cu is lower than that of sGr/Cu, highlighting the importance of graphene size regulation and dispersion optimization.

Electrochemical impedance spectroscopy (EIS) is a non-destructive technique widely used to evaluate corrosion behavior over a broad frequency range [28,29,30]. Figure 5b–d present the Bode plots, Nyquist plots, and corresponding equivalent electrical circuit fitting results for the three copper foils in 3.5 wt.% NaCl solution. In the EEC model, R_s_ represents the solution resistance; the constant phase element describes the capacitive behavior of the corrosion interface; R_ct_ represents the charge transfer resistance associated with interfacial electrochemical reactions; and W represents the Warburg impedance related to diffusion processes near the electrode surface. The fitted electrochemical parameters are listed in Table 3.

From the Bode plots (Figure 5b), the low-frequency impedance modulus of sGr/Cu is higher than those of Cu and Gr/Cu. Furthermore, sGr/Cu exhibits the highest phase angle peak, suggesting enhanced stability of the interfacial protective layer and improved corrosion resistance. The radius of the capacitive arcs in the Nyquist plots follows the order: sGr/Cu > Gr/Cu > Cu. A larger capacitive arc radius generally corresponds to a higher interfacial charge transfer resistance (R_ct_), indicating slower interfacial corrosion reactions [31]. The fitted results show that the R_ct_ value of sGr/Cu reaches 3789 Ω·cm^2^, which is higher than those of Cu (1640 Ω·cm^2^) and Gr/Cu (2922 Ω·cm^2^). This increase in charge transfer resistance indicates that the electrochemical corrosion process at the sGr/Cu interface is more effectively inhibited. The improved electrochemical performance of sGr/Cu can be attributed to the synergistic effects of graphene dispersion, surface morphology optimization, and interfacial structure regulation. The uniform distribution of size-reduced graphene within the copper matrix contributes to the formation of a more continuous barrier network, increasing the resistance to corrosive species penetration. Meanwhile, the reduced surface defects and optimized copper texture further suppress localized corrosion reactions. Consequently, the interfacial charge transfer process is inhibited, resulting in enhanced corrosion resistance of the sGr/Cu composite foil. These results support the enhanced corrosion resistance mechanism of sGr/Cu from the perspective of electrochemical kinetics.

### 2.6. Surface Wettability Analysis

**Figure 6:**
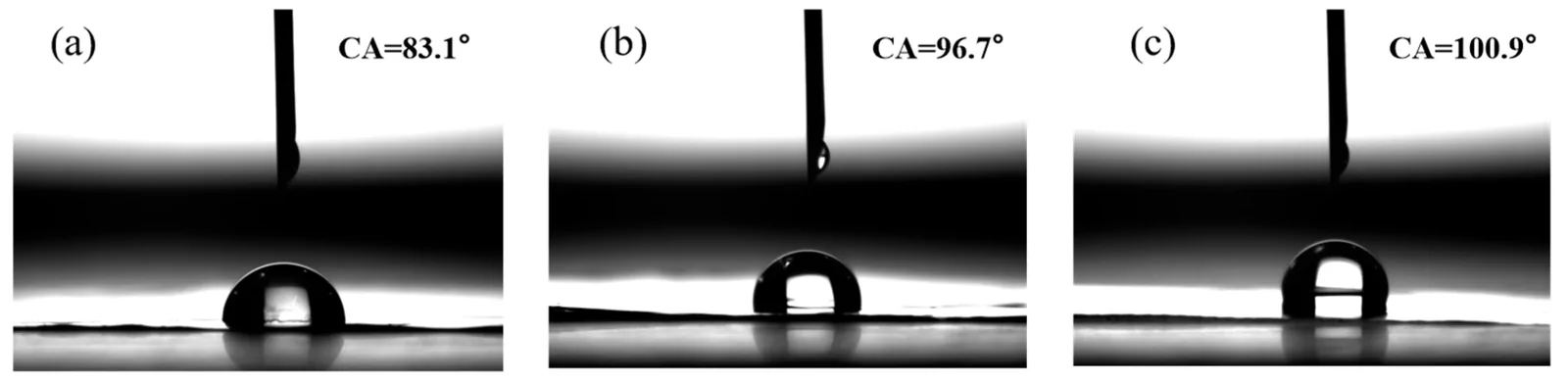
Water contact angle measurements of the three copper foils: (**a**) Cu, (**b**) Gr/Cu, and (**c**) sGr/Cu.

The surface wettability of the three copper foils was evaluated by measuring the static water contact angle, and the corresponding results are shown in Figure 6. The water contact angles of Cu, Gr/Cu, and sGr/Cu are 83.1°, 96.7°, and 100.9°, respectively. The contact angle gradually increases after graphene incorporation, indicating enhanced surface hydrophobicity [14]. This increase may be attributed to the hydrophobic characteristics of graphene and its improved distribution on the copper foil surface. Notably, sGr/Cu exhibits the highest contact angle, suggesting that graphene size reduction improves graphene dispersion and surface coverage, thereby enhancing the water repellency of the composite foil. The increased contact angle indicates reduced surface affinity toward water, which may decrease the retention of aqueous corrosive species and contribute to improved corrosion resistance. Combined with the reduced surface roughness observed by SEM, the enhanced hydrophobicity of sGr/Cu may provide an additional contribution to corrosion protection.

### 2.7. XPS Interfacial Bonding Analysis

**Figure 7:**
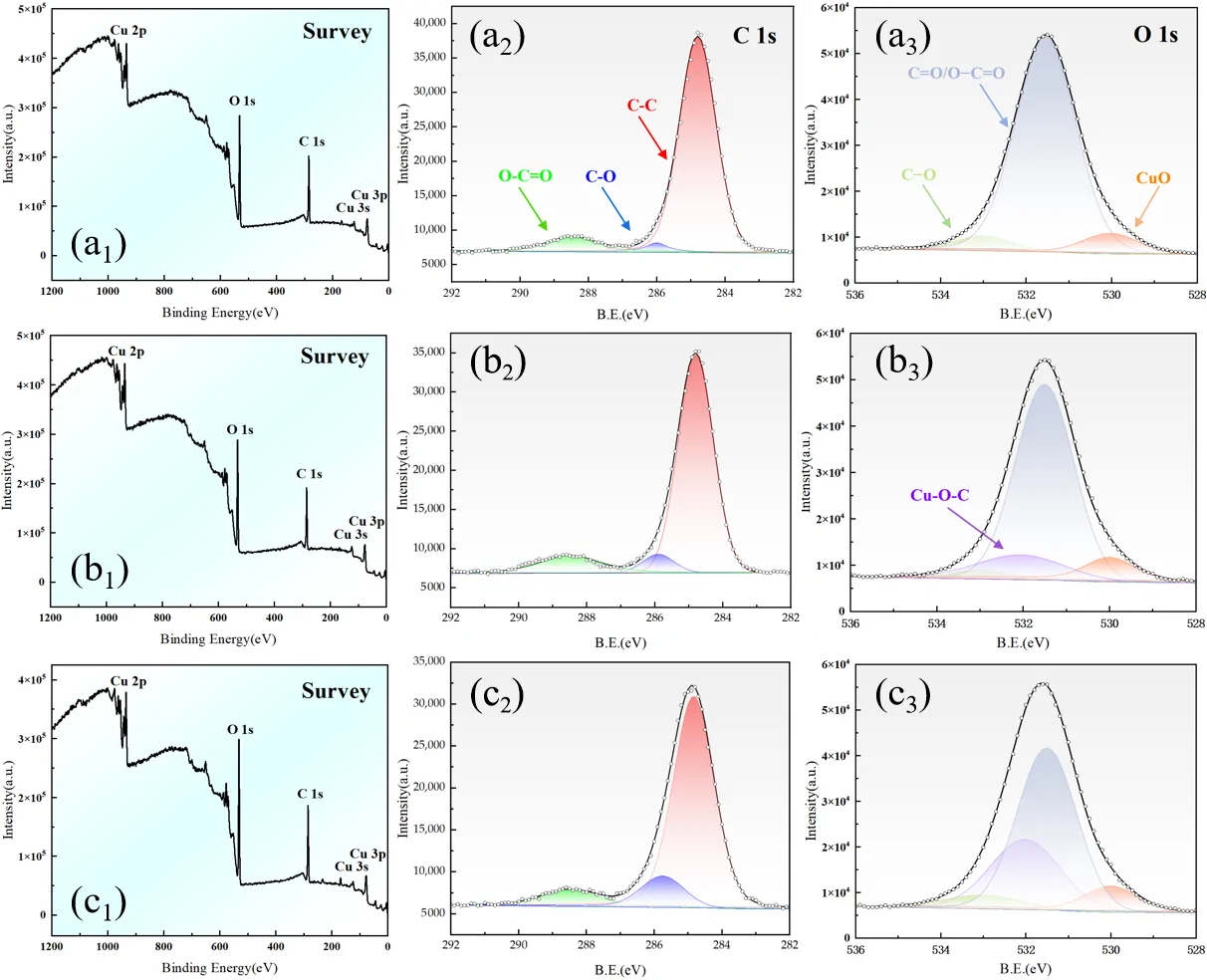
XPS spectra of the three copper foils: (**a_1_**–**a_3_**) Cu, (**b_1_**–**b_3_**) Gr/Cu, and (**c_1_**–**c_3_**) sGr/Cu.

Figure 7 presents the X-ray photoelectron spectroscopy (XPS) analysis results of the three copper foils, aiming to investigate the surface chemical states and potential interfacial interactions of the copper foils. The survey spectra Figure 7(a_1_,b_1_,c_1_) show characteristic peaks assigned to the C 1s, O 1s and Cu 2p orbitals, together with several minor impurity peaks most likely introduced during sample storage, which confirms the elemental composition of each sample.

The high-resolution C 1s spectra can be deconvoluted into three characteristic peaks corresponding to C-C (284.8 eV), C-O (286.0 eV) and O-C=O (288.6 eV) bonding configurations [32]. These spectra can reflect the chemical interactions between graphene and copper. After graphene is incorporated into the copper matrix, the C 1s peaks of both Gr/Cu and sGr/Cu shift slightly toward higher binding energies, indicating that the electronic environment of graphene is altered owing to its interaction with the copper matrix [33]. Notably, sGr/Cu exhibits a larger peak shift, representing stronger interfacial interactions between graphene and copper. The enhanced interfacial interaction can be attributed to the size reduction in graphene, which increases the interfacial contact area between graphene and the copper matrix.

The high-resolution O 1s spectra further verify the interfacial chemical bonding: the pure copper sample without graphene only displays characteristic peaks corresponding to CuO (530 eV), C-O (533.0 eV) and C=O/O-C=O (531.5 eV) [33,34]. In contrast, both composite copper foils (Gr/Cu and sGr/Cu) show a distinct peak at 532.0 eV, which is assigned to Cu-O-C covalent bonds. Such Cu-O-C bonding can effectively fill the micro-gaps between graphene sheets and the copper matrix and block the penetration pathways of corrosive media along the interface. Therefore, the corrosion resistance of the sGr/Cu sample is significantly improved.

## 3. Materials and Methods

### 3.1. Reagents and Materials

Copper sulfate pentahydrate (CuSO_4_·5H_2_O), hydrochloric acid (HCl), sodium poly(dithiodipropane sulfonate) (C_6_H_15_NaO_6_S_4_), polyethylene glycol ((C_2_H_4_O)_11_H_2_O), gelatin (C_6_H_12_O_6_), sulfuric acid (H_2_SO_4_), and graphene (Gr) were all purchased from Shanghai Aladdin Biochemical Technology Co., Ltd. (Shanghai, China). All reagents were used directly without further purification. According to the supplier’s specifications, the graphene (Gr) is few-layer graphene with fewer than 5 layers and a lateral size of 2–5 μm. The small-size graphene (sGr) has an average lateral size of approximately 300 nm.

### 3.2. Preparation of Electrolytic Copper Foils

Electrolytic copper foils (with a thickness of 8 μm) were prepared using a direct current (DC) electrodeposition method. The base electrolyte formulation was: Cu^2+^ 64 g/L, H_2_SO_4_ 120 g/L, Cl^−^ 30 mg/L, SPS 2.5 mg/L, PEG 4.0 mg/L, and gelatin 8.0 mg/L. On this basis, three groups of comparative samples were prepared by adding different forms of graphene to the base electrolyte: (1) Pure copper foil without graphene addition (denoted as Cu); (2) Composite copper foil with pristine graphene addition (denoted as Gr/Cu); (3) Composite copper foil with small-size graphene, obtained by ball milling treatment (denoted as sGr/Cu). The small-size graphene was prepared using a planetary ball mill at a rotation speed of 300 r/min for 50 h. The cathode for electrodeposition was a polished titanium plate, and the anode was a ruthenium-iridium titanium plate, with an inter-electrode distance of 10 mm. During electrodeposition, the current density was maintained at a constant 20 A·dm^−2^, and the electrolyte temperature was controlled at 40 ± 0.5 °C. After electrodeposition, the obtained electrolytic copper foil was peeled from the cathode and thoroughly rinsed with deionized water to remove residual plating solution. Subsequently, the copper foil was dried in air and stored in a constant-temperature drying oven. The side peeled from the titanium substrate was designated as the shiny side (S-side), and the other side as the matte side (M-side). Surface roughness of each copper foil was measured using a roughness tester (PS10, Mahr). The probe traversed laterally on the matte side of the copper foil over a length of 4.8 mm. Each sample was tested five times, and the average value was used for data analysis, with the error calculated.

### 3.3. Microstructural Characterization

The microstructure and elemental distribution on the matte side of the copper foils were characterized using a field-emission scanning electron microscope (SEM, ZEISS GeminiSEM 460, Carl Zeiss Microscopy GmbH, Jena, Germany) equipped with an energy-dispersive spectrometer (EDS, Oxford Xplore 30, Oxford Instruments plc, Abingdon, UK). The SEM acceleration voltage was 20 kV. Phase identification was performed using an X-ray diffractometer (XRD, Bruker D8 Advance, Bruker AXS SE, Karlsruhe, Germany) with Cu Kα radiation (λ = 0.15406 nm) at a scanning rate of 5°/min. The chemical states of elements on the copper foil surface, particularly the carbon bonding configurations and the interfacial interactions between graphene and the copper matrix, were analyzed using X-ray photoelectron spectroscopy (XPS, Thermo Fisher VG Multilab 2000, Thermo VG Scientific, East Grinstead, UK). All XPS spectra were charge-referenced to the C-C component at 284.8 eV. Shirley background subtraction was applied, and peak deconvolution was carried out using a mixed Gaussian-Lorentzian function.

### 3.4. Electrochemical Performance Testing

All electrochemical experiments were performed using a CHI 660E (Shanghai Chenhua Instrument Co., Ltd., Shanghai, China) electrochemical workstation and a standard three-electrode system. The copper foils peeled off from the electrodeposition experiments were directly used as the working electrode. Insulating sealant was applied for sample sealing, so that only a fixed frontal area of the copper foil was exposed to the electrolyte. Electrochemical impedance spectroscopy (EIS) measurements were carried out at the open-circuit potential (OCP). Data acquisition was started after the open-circuit potential of the working electrode became stable. ZView3.1 software was adopted to perform equivalent-circuit fitting for the EIS experimental data. Contact angle measurements were performed according to the national standard GB/T 22638.4-2016 [35], using a Dataphysics OCA 20 (DataPhysics Instruments GmbH, Filderstadt, Germany) optical contact angle measuring instrument with deionized water as the test liquid (droplet volume 3.5 μL). Five different positions on each sample were tested, and the average value was calculated.

## 4. Conclusions

In this study, size-reduced graphene was incorporated into ultra-thin copper foils through direct-current electrodeposition to improve their corrosion resistance. The results demonstrate that graphene incorporation, particularly size-reduced graphene incorporation, improves the surface morphology and corrosion resistance of copper foils. SEM analysis revealed that the incorporation of size-reduced graphene reduced surface defects and surface roughness, resulting in a more uniform and compact copper foil surface. XRD analysis showed that size-reduced graphene influenced copper crystallographic evolution and promoted preferential growth of the (220) plane, which may contribute to improved corrosion resistance. Electrochemical corrosion tests demonstrated that sGr/Cu exhibited enhanced corrosion resistance, with the lowest corrosion current density of 3.781 × 10^−5^ A·cm^−2^ and the highest charge transfer resistance of 3789 Ω·cm^2^. The improved corrosion resistance was attributed to the synergistic effects of graphene-induced barrier effects, reduced surface roughness, enhanced surface hydrophobicity, and strengthened graphene/copper interfacial interactions. XPS analysis further suggested the presence of Cu–O–C-related interfacial interactions, which may enhance the bonding between graphene and the copper matrix. Overall, this work provides an effective strategy for fabricating corrosion-resistant ultra-thin copper foils through graphene size regulation and interface optimization.

## Figures and Tables

**Table 1:** Relative texture coefficients of the three samples.

Sample	(111) Plane (%)	(200) Plane (%)	(220) Plane (%)
Cu	36.6	27.6	35.8
Gr/Cu	15.3	16.5	68.2
sGr/Cu	7.6	8.7	83.7

**Table 2:** Corrosion potential, corrosion current density, and corrosion rate of the three samples.

Sample	Ecorr (V, vs. Sce)	Icorr (A·cm^−2^)	Vcorr (mm·a^−1^)
Cu	−0.229	9.594 × 10^−5^	1.113
Gr/Cu	−0.133	4.275 × 10^−5^	0.496
sGr/Cu	−0.099	3.781 × 10^−5^	0.438

**Table 3:** Equivalent circuit fitting results of the three samples.

Sample	R_s_ Ω·cm^2^	C_t_ F·m^−2^	C_p_ F·m^−2^	R_ct_ Ω·cm^2^	W_r_ Ω·cm^2^	W_t_ Ω·cm^2^	W_p_ Ω·cm^2^
Cu	5.15	2.01 × 10^−4^	0.673	1640	2762	121.9	0.471
Gr/Cu	4.61	1.82 × 10^−4^	0.722	2922	3164	142.8	0.402
sGr/Cu	4.78	1.66 × 10^−4^	0.823	3789	2587	128.1	0.328

## Data Availability

All data supporting the findings of this study are included within the article.

## Supplementary Materials

The following supporting information can be downloaded at: https://www.mdpi.com/article/10.3390/molecules31183341/s1, Figure S1: (a) TEM image of pristine graphene (Gr), (b) fast Fourier transform (FFT), (c) TEM image of small-size graphene (sGr), (d) the corresponding magnified local view of sGr; Figure S2: Raw-data Nyquist plot; Table S1: The pit size and areal density of corrosion pits for the three samples after corrosion; Table S2: Equivalent-circuit fitting parameter error results of the three samples.
