# Supplementary material for: Small-Size Graphene-Enabled Corrosion-Resistant Ultra-Thin Copper Foils Fabricated by Direct-Current Electrodeposition
Source: Molecules. 2026 Sep 20;31(18):3341. doi: 10.3390/molecules31183341 (PMC13610526; doi:10.3390/molecules31183341)
Supplement: Supplementary file 1 [file molecules-31-03341-s001.zip › molecules-4548888-supplementary.pdf]

Supplementary Material

Small-Size Graphene Enabled Corrosion-Resistant Ultra-Thin Copper Foils Fabricated by Direct-Current Electrodeposition

Zheng Yu [1,2], Hanyang Zhao [1], Junpeng Li [3], Jinzhong Li [3], Lixu Zhu [3], Xiaowen Zhang [1], Wenjuan Niu [2], Feng Wang [1], Kewang Zheng [1] and Fei Zhong [1,*]

[1] School of Chemistry and Materials Science, Hubei Engineering University, Xiaogan 432000, China;

[2] Key Laboratory of Agricultural Equipment in Mid-Lower Yangtze River, Ministry of Agriculture, College of Engineering, Huazhong Agricultural University, Wuhan 430070, China

[3] Dongguan Yichuang Surface Treatment Technology Co., Ltd, Dongguan 532000, China

* Correspondence: zhong4536188@hbeu.edu.cn

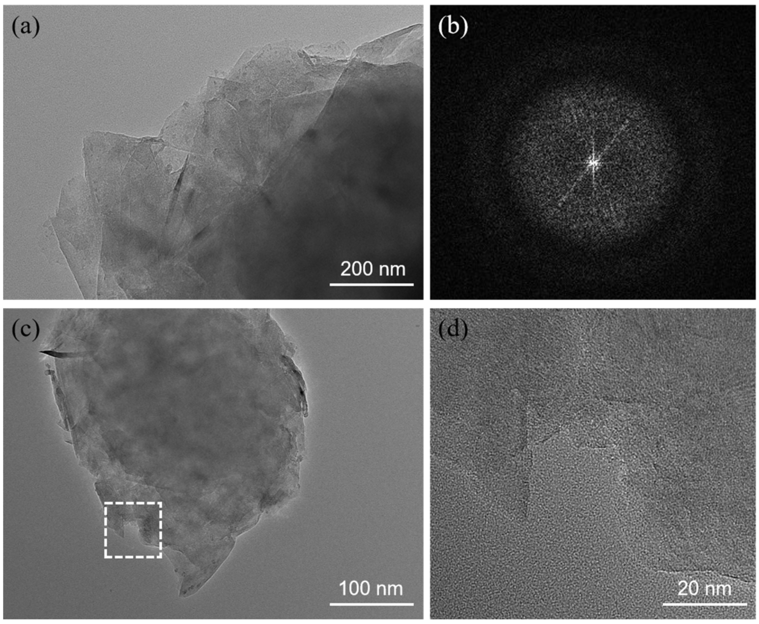

Figure S1. (a) TEM image of pristine graphene (Gr), (b) fast Fourier transform (FFT), (c) TEM image of small-size graphene (sGr), (d) the corresponding magnified local view of sGr

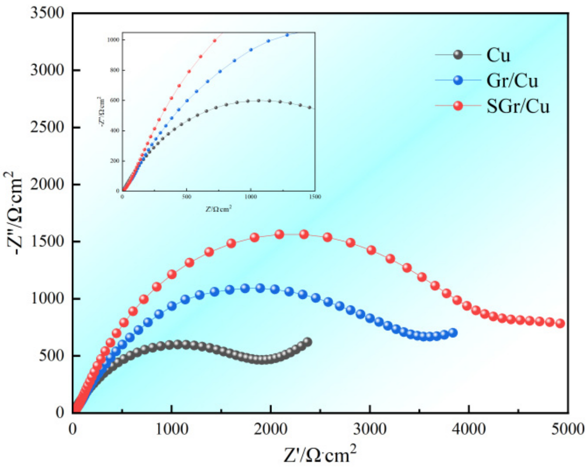

Figure S2. Raw-data Nyquist plot

Table S1. The pit size and areal density of corrosion pits for the three samples after corrosion.

Sample	Average diameter（μm）	areal density of corrosion pits (%)
Cu	9.5428×10^{-2}	9.724
Gr/Cu	7.7605×10^{-2}	4.872
sGr/Cu	6.1391×10^{-2}	3.138

Table S2. Equivalent-circuit fitting parameter error results of the three samples.

**Sample**	**R_s** (Error%)	**C_t** (Error%)	**C_p** (Error%)	**R_{ct}** (Error%)	**W** (Error%)
Cu	2.268	1.797	0.802	2.122	7.3037
Gr/Cu	1.478	1.333	0.704	2.047	1.1412
sGr/Cu	9.070	2.027	0.970	2.781	2.022
